# Citrate-Stabilized Amorphous Calcium Phosphate Nanoparticles as an Effective Adsorbent for Defluorination

**DOI:** 10.3390/nano15080621

**Published:** 2025-04-18

**Authors:** Ruojiao Su, Miaomiao Wang, Yuwei Jiang, Shuang Zhang, Junjun Tan

**Affiliations:** 1 Hubei Provincial Key Laboratory of Green Materials for Light Industry, Hubei University of Technology, Wuhan 430068, China; 102200427@hbut.edu.cn (R.S.); 102200424@hbut.edu.cn (M.W.); 102210519@hbut.edu.cn (Y.J.); 102300397@hbut.edu.cn (S.Z.); 2 Collaborative Innovation Center of Green Light Weight Materials and Processing, Hubei University of Technology, Wuhan 430068, China; 3 School of Materials and Chemical Engineering, Hubei University of Technology, Wuhan 430068, China

**Keywords:** amorphous calcium phosphate, sodium citrate, fluoride removal, hydroxyapatite

## Abstract

Amorphous calcium phosphate (ACP), one of the most important calcium–phosphorus compounds, is widely used in dentistry, orthopedics, and medicine, but is rarely reported for fluoride removal from water. In view of this, sodium citrate-stabilized amorphous calcium phosphate (Cit-ACP) and Cit-ACP calcinated at different temperatures were successfully prepared for fluoride removal. The results showed that the adsorption data of the Cit-ACP sample could be well described by the Langmuir model, and the adsorption kinetic followed the pseudo-second-order model. The maximum adsorption capacity was 27.48 mg/g at pH 7.0 when the fluoride concentration is 100 mg/L. The thermodynamic parameters suggested that the adsorption of fluoride was a spontaneous endothermic process. The XRD, XPS, and Zeta potential analysis of the Cit-ACP sample before and after fluoride removal revealed that, owing to the core–shell structure of the Cit-ACP nanoparticles, the fluoride ions in solution and the calcium ions in shell layer of the Cit-ACP nanoparticles co-promoted the transformation of the core of the Cit-ACP nanoparticles into fluorapatite. Given the simplicity of its preparation and effectiveness of its fluoride removal properties, Cit-ACP would be a potentially economical, efficient, and biocompatible adsorbent for fluoride removal.

## 1. Introduction

Fluorine is an essential trace element for human beings and animals. The presence of a certain amount of fluoride in drinking water has beneficial effects on human health, such as preventing tooth decay and strengthening tooth enamel [1]. However, if the fluoride concentration exceeds 1.5 mg per liter, this will damage human health and lead to many diseases such as dental/skeletal fluorosis, fetal cerebral function, and neurotransmitters [2,3].

Given the health hazards of excess fluoride in water, many technologies have been developed to remove fluoride [3]: electrocoagulation [4,5,6], precipitation [7,8], ion exchange [9,10], membrane techniques [11,12], and adsorption [13,14]. Of these techniques, adsorption has proven to be an effective, environmentally friendly, and economical method for the removal of excess fluoride from drinking water [2,15]. Different adsorbents have been successfully utilized for fluoride removal, such as hydroxyapatite [16,17], alumina-based adsorbents [18,19], activated carbon [20,21], ion exchange resins [22], biopolymer-based composites [23,24], clay-based materials [25,26], and mixed oxides [27,28]. However, to date, some problems associated with their applications are still unresolved, such as low adsorption capacities and poor selectivity. 

Amorphous calcium phosphate (ACP) [29,30,31], often found in the early stages of biological hard tissue formation or synthetic hydroxyapatite, is often considered to be an intermediate stable calcium–phosphorus compound with the typical chemical formula [M_u_Ca_3_(HPO_4_)_3v_ (PO_4_)_3y_]·zH_2_O [32]. Due to its excellent bioactivity, biocompatibility, bioabsorbability, and non-toxicity, ACP is an important biomaterial that is of significant interest in dentistry, orthopedics, and other biomedical fields [33,34,35,36,37].

Under the right temperature, pH, and humidity conditions, ACP can be rapidly converted to other thermodynamically more stable calcium–phosphorus compounds such as hydroxyapatite [38,39,40]. In order to prepare ACP, biomolecules or organic small molecules are often used to assist in its formation, such as PEG2000, sodium citrate, casein, etc. [34,41,42]. Of these, sodium citrate is one of the most popular [43]. Sodium citrate-stabilized amorphous calcium phosphate (Cit-ACP) has a high specific surface area of 200–330 square meters per gram, compared to amorphous calcium phosphate (blank-ACP) which has a specific surface area of only 60–160 square meters per gram. Cit-ACP dry powder can be stored at room temperature for up to four years without any change. In contrast, blank-ACP powders spontaneously convert to crystalline HA within a few hours under ambient conditions. Moreover, the sodium citrate-stabilized ACP nanoparticles are very sensitive to fluoride ions and can be rapidly converted to fluorapatite in a solution environment where fluoride ions are present [44].

Combining the above characteristics, it is reasonable to infer that Cit-ACP nanoparticles may be an efficient fluoride adsorbent. So far, there are few reports on Cit-ACP nanoparticles in fluoride removal compared to the numerous reports on hydroxyapatite in fluoride removal. In this work, the removal of fluoride by Cit-ACP nanoparticles as an adsorbent was systematically investigated for the first time. The fluoride removal capacity, the adsorption thermodynamics, and the kinetics of Cit-ACP were studied. Then, the effects of pH, initial fluoride concentration, and co-existing anions on the adsorption performance of Cit-ACP were investigated. Finally, the adsorption mechanism of Cit-ACP was illustrated. 

## 2. Materials and Methods

### 2.1. Materials

Calcium chloride dihydrate (CaCl_2_·2H_2_O, purity ≥ 99.0%), sodium phosphate tribasic dodecahydrate (Na_3_PO_4_·12H_2_O, purity ≥ 98.0%), sodium citrate tribasic dihydrate (C_6_H_5_Na_3_O_7_·2H_2_O, purity ≥ 99.0%), sodium fluoride (NaF, purity ≥ 97.0%), and sodium hydroxide (NaOH, purity ≥ 97.0%) were purchased from Shanghai Aladdin Reagent Co., Ltd. (Shanghai, China) All chemicals were used as received without further purification. Deionized water was used throughout the study.

### 2.2. Preparation of Adsorbent

In a typical synthesis, an aqueous solution of sodium citrate (0.02 mol, 30 g H_2_O) was slowly added to an aqueous solution of calcium chloride (0.02 mol, 30 g H_2_O) with continuous stirring for 30 min. Then, an aqueous solution of sodium phosphate (0.012 mol, 40 g H_2_O) was slowly added to the mixture with vigorous stirring for 60 min. Afterwards, the resulting product was purified by a two-round centrifugation–wash process with deionized water. Finally, the precipitate was freeze-dried, and the resulting powder was used as an adsorbent, labeled as sample Cit-ACP-25. In order to compare the adsorption properties, the sample Cit-ACP-25 was subjected to a two-hour calcination process at 200, 400, and 600 °C. The resulting adsorbents were labeled as sample Cit-ACP-200, Cit-ACP-400, and Cit-ACP-600, respectively. The preparation process is shown schematically in Figure S1.

### 2.3. Batch Adsorption Experiments

In order to evaluate the adsorption performance of the prepared adsorbent on fluoride ions in water, adsorption experiments were carried out. The sodium fluoride stock solution was obtained by dissolving 4.421 g of sodium fluoride in 1000 mL of deionized water, at which the concentration of fluoride ions was 2000 mg/L. The test solutions with different fluoride ion concentrations required for the experiments were obtained by diluting the sodium fluoride stock solution.

The adsorption isotherm was measured at a pH of 7.0 and an adsorbent addition of 1 g/L. A total of 30 mg of adsorbent was added to a polyethylene centrifuge tube containing 30 mL of a specific concentration of sodium fluoride test solution (5 to 100 mg/L) and then the flasks were shaken at 100 rpm in a shaker at three different temperatures, viz., 25, 35, and 45 °C for 24 h to reach adsorption equilibrium. After the completion of adsorption, the solution was centrifuged at 8000 rpm for 5 min and its supernatant was used by a fluoride ion selective electrode (PF-2-01, Leici, Shanghai, China) for the determination of fluoride ion concentration. The relationship between the fluoride ion standard solution concentration and the electrode potential values is shown in Figure S2.

All measurements were carried out at 25 °C and the adsorption capacity (q_e_, mg/g) was calculated according to the following equation [45]:

(1) $$q_{e}=\frac{(C_{0}-C_{e})V}{m}$$

where *C*_0_ and *C_e_* are the initial and final equilibrium concentrations (mg/L) of fluoride in solution, respectively; *V* is the volume of sodium fluoride test solution (L); and *m* is the mass of adsorbent added (g).

In the kinetic experiments, a series of polyethylene centrifuge tubes containing sodium fluoride test solution (10 mg/L fluoride ion concentration) were added with 30 mg of adsorbent and quickly placed in a constant temperature shaker at 100 rpm and 25 °C. When a specific contact time was reached, one of the centrifuge tubes was quickly removed for centrifugation and the fluoride ion concentration of the test solution after adsorption was measured.

The effect of the initial pH of sodium fluoride test solution on the adsorption performance of the adsorbent was carried out by changing the initial pH of the sodium fluoride test solution using 0.1 M hydrochloric acid and 0.1 M NaOH, and the pH adjustment range was from 3 to 11. In this experiment, the initial fluoride concentration was 10 mg/L, the adsorption temperature was 25 °C, adsorption time was 24 h, and the adsorbent addition was 1 g/L.

The effect of co-existing ions on the adsorption performance of the adsorbent was carried out by adding different types (chloride, phosphate, sulfate, and bicarbonate ions) and different concentrations (0.3 mM, 0.6 mM, 1.2 mM, 2.4 mM) of co-existing ions to the sodium fluoride test solution. In this experiment, the initial fluoride concentration was 10 mg/L and the adsorbent dose was 1 g/L.

The experimental data were fitted using the Langmuir and Freundlich model with the model equation shown below [46]:

(2) $$\frac{C_{e}}{q_{e}}=\frac{1}{K_{L}q_{m}}+\frac{C_{e}}{q_{m}}$$

(3) $$\ln q_{e}=\ln K_{F}+\frac{1}{n}\ln C_{e}$$

where *q_e_* is the adsorption capacity at adsorption equilibrium in mg/g; *C_e_* is the equilibrium concentration of fluoride ions in mg/L; *q_m_* is the maximum adsorption capacity of the adsorbent for fluoride ions in mg/g; *K_L_* is the Langmuir’s constant of the adsorption process in L/mg; *K_F_* is the Freundlich’s constant of the adsorption process; and 1/n is the adsorption strength.

The equations for each of the two kinetic models are as follows [47]:

(4) $$\ln(q_{e}-q_{t})=\ln q_{e}-k_{1}t$$

(5) $$\frac{t}{q_{t}}=\frac{1}{k_{2}q_{e}^{2}}+\frac{t}{q_{e}}$$

where *q_t_* is the adsorption capacity of fluoride ion at any time *t*; *q_e_* is the adsorption capacity of fluoride ions at the time of reaching adsorption equilibrium, in mg/g; *k*_1_ is the rate constant for the pseudo-first-order reaction in min^−1^; *k*_1_ is the rate constant for the pseudo-first-order reaction in min^−1^; and *k*_2_ is the rate constant for the pseudo-second-order reaction in g/(mg·min).

### 2.4. Characterization

#### 2.4.1. Wide-Angle X-Ray Diffraction (XRD)

Powder X-ray diffraction was performed in Bragg–Brentano geometry with an Empyrean nano-X-ray diffractometer (Panalytical, Almelo, The Netherlands) equipped with a Cu Kα radiation (0.15406 nm) source. Scans were performed from 10° to 80° with a scan rate of 6°/min.

#### 2.4.2. Field-Emission Scanning Electronic Microscopy (SEM)

The particle morphology of the samples was examined by scanning electron microscopy (SEM; Hitachi SU-8010, Hitachi High Technologies, Tokyo, Japan) using a secondary electron detector at 0° tilt angle and a working distance of 4 mm. Prior to SEM observation, the samples were sputter-coated with gold for 30 s using an MSP-2S magnetron sputter (Hitachi High Technologies, Tokyo, Japan) to enhance conductivity.

#### 2.4.3. Zeta Potential Measurement

The zeta potential of Cit-ACP nanoparticles was measured using a Zetasizer Nano ZS90 (Malvern Instruments Ltd., Malvern, UK) at varying pH values. The nanoparticle concentration in solution was maintained at 0.5 wt.% for all measurements. The data were recorded and analyzed using Dispersion Technology Software v. 5.0 (Malvern Instruments). The pH of sample solutions was adjusted using 0.1 M NaOH or 0.1 M HCl solution, which was determined with a PB-10 pH meter (Sartorius AG, Goettingen, Germany) at 25 °C. No additional electrolytes were added during the measurement.

#### 2.4.4. X-Ray Photoelectron Spectroscopy (XPS)

X-ray photoelectron spectroscopy analyses of the samples were collected on a thermos ESCALAB 250 X-ray photoelectron spectrometer with Al Ka radiation.

#### 2.4.5. Brunauer–Emmett–Teller Measurement (BET)

The Brunauer–Emmett–Teller specific surface and pore structure of the products were calculated from N_2_ adsorption–desorption isotherms at 77 K, and the plot of the pore size distribution was determined using the Barett–Joyner–Halenda (BJH) method from the desorption branch of the isotherm by an ommishop 100CX surface area analyzer (Coulter Inc., Brea, CA, USA).

## 3. Results and Discussion

### 3.1. Characterization of Adsorbents

**Figure 1:**
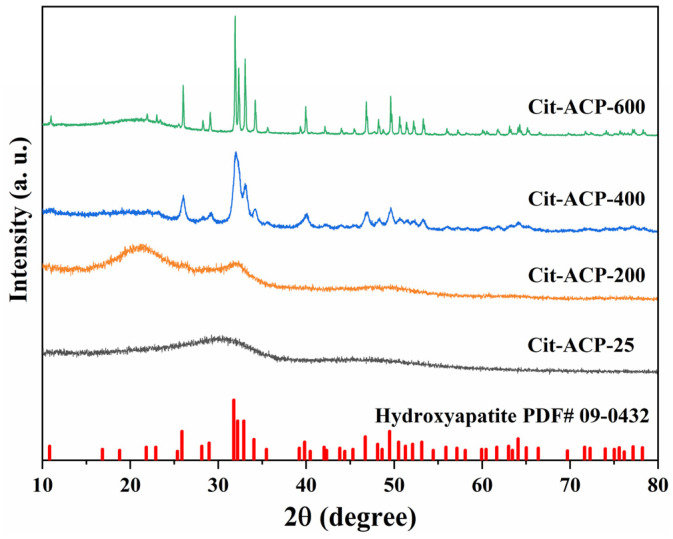
XRD pattens of the prepared samples.

The characteristics of adsorbents, such as particle size, specific surface area, crystallinity, and other relevant information, are useful in understanding the adsorption behavior of adsorbents. Figure 1 shows the XRD patterns of the prepared samples. The XRD patterns of both samples Cit-ACP-25 and Cit-ACP-200 do not have typical characteristic diffraction peaks, indicating that the samples are amorphous calcium phosphate. The XRD patterns of the samples Cit-ACP-400 and Cit-ACP-600 display the following diffraction peaks at 26.0°, 32.1°, 34.1°, 39.9°, 46.8°, 49.6°, 53.3°, and 64.1° which are correlated to the (h k l) indices (0 0 2), (2 1 1), (2 0 2), (3 1 0), (2 2 2), (2 1 3), (0 0 4), and (3 0 4) planes, respectively. All reflections are characteristics of the hexagonal phase of HA according to the standard data (JCPDS No. 09-0432) [48] and reported studies [49].

In addition, as the sintering temperature was increased from 400 to 600 °C, the profiles of the diffraction peaks of the samples became more and more complete and sharp, indicating that the increase in the sintering temperature not only transformed the samples from amorphous calcium phosphate to crystallized hydroxyapatite but also increased the crystallinity of the product.

**Figure 2:**
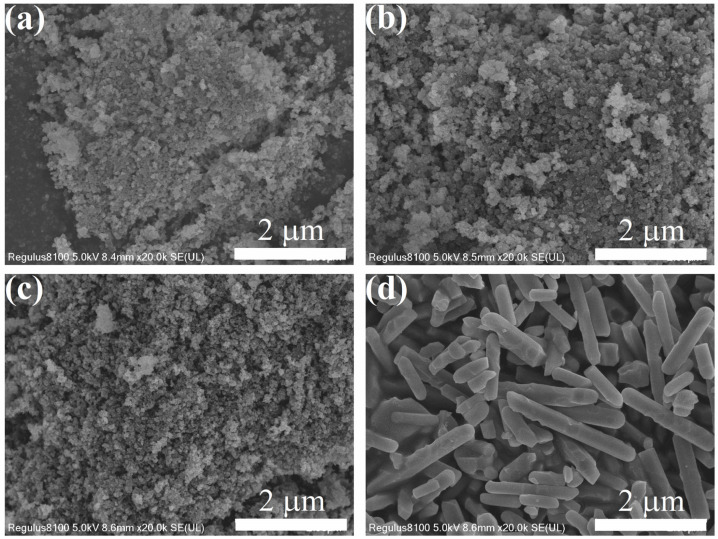
SEM images of the prepared samples: (**a**) Cit-ACP-25, (**b**) Cit-ACP-200, (**c**) Cit-ACP-400, and (**d**) Cit-ACP-600.

Figure 2 are SEM images of the prepared samples. When not sintered, sample Cit-ACP-25 presents as spherical particles with an average diameter around 33 nm. When the sintering temperature was increased to 400 °C, the morphology of the sample particles did not change significantly, but the size increased slightly, e.g., 50 nm (Cit-ACP-200) or 26 nm (Cit-ACP-400). When the sintering temperature is 600 °C, the morphology of the sample particles changes from spherical to rod-like, and the size of the particles increases dramatically, with an average length of 1.2 µm and an average diameter of 311 nm.

The average crystallite size of the synthesized nanoparticles was estimated using the Scherrer equation:

(6) $$D=\frac{K\lambda }{\beta \cos\theta }$$

where *D* is the crystallite size (nm), *K* is the Scherrer constant (0.90 for spherical particles), *λ* is the X-ray wavelength (Cu Kα radiation, 0.15406 nm), *β* is the full width at half maximum (FWHM) of the diffraction peak in radians, and *θ* is the Bragg angle. The (002) diffraction peak at 2*θ* = 26.0°. Substituting the values, the crystallite sizes of samples Cit-ACP-400 and Cit-ACP-600 were calculated to be approximately 6.04 nm and 49.52 nm, respectively.

Combined with the XRD results and SEM observation, it can be deduced that when the sintering temperature is below 200 °C, it is mainly attributed to the changes brought about by the loss of crystalline water from amorphous calcium phosphate. When the sintering temperature rises above 400 °C, the amorphous calcium phosphate is transformed into crystallized hydroxyapatite nanoparticles and continues to increase with temperature, accompanied by austenitic ripening and recrystallization.

**Figure 3:**
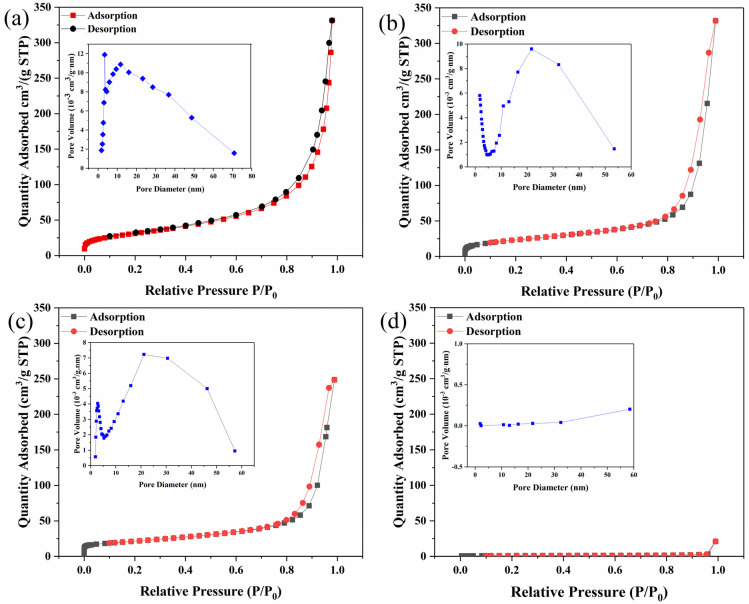
Nitrogen adsorption–desorption isotherms of the prepared samples: (**a**) Cit-ACP-25, (**b**) Cit-ACP-200, (**c**) Cit-ACP-400, and (**d**) Cit-ACP-600. The inset is the pore size distribution curve of the prepared samples.

In order to analyze the specific surface area and pore structure distribution of the samples, the samples were subjected to BET analysis as shown in Figure 3. The nitrogen adsorption–desorption isotherms for all samples were typical of type IV and H4 hysteresis loops, indicating the presence of mesoporous structures. The specific surface areas of samples Cit-ACP-25, Cit-ACP-200, Cit-ACP-400, and Cit-ACP-600 were 113.32, 84.70, 75.50, and 3.38 m^2^/g, and the average pore sizes were 17.69, 24.58, 23.18, and 55.13 nm, respectively. The pore distribution curves further confirm the presence of mesoporous structures in the samples.

From the above results, it can be seen that the samples without sintering have the highest specific surface area. As the sintering temperature increases, the specific surface area of the samples gradually decreases. Especially when the sintering temperature is 600 °C, the specific surface area of the sample is only a third of that of the unsintered sample, which is consistent with the SEM observation.

### 3.2. Fluoride Ion Adsorption Behavior of the Samples

#### 3.2.1. Adsorption of Time Dependence

**Figure 4:**
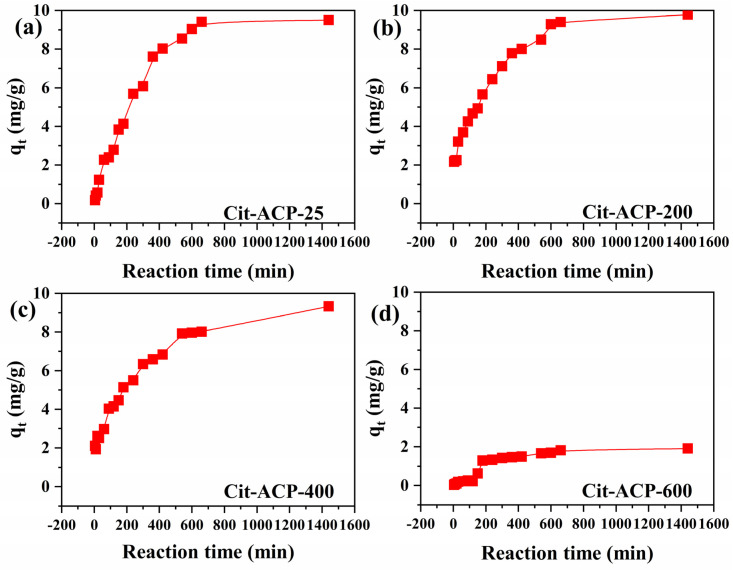
Adsorption kinetics of fluoride on prepared samples (initial fluoride concentration: 10 mg/L; adsorbent dose: 1.0 g/L; temperature: 25 °C; pH: 7.0). (**a**) Cit-ACP-25, (**b**) Cit-ACP-200, (**c**) Cit-ACP-400, and (**d**) Cit-ACP-600.

Figure 4 shows the adsorption curves of the samples with time at an initial fluoride ion concentration of 10 mg/L. The adsorption capacity of samples Cit-ACP-25, Cit-ACP-200, Cit-ACP-400, and Cit-ACP-600 was rapidly increased in the time ranges of 360, 360, 300, and 120 min, respectively, and then approached the adsorption equilibrium at 660, 600, 540, and 200 min, respectively.

In order to better understand the effect of adsorption time on fluoride removal, the experimental data were linearly fitted according to pseudo-first-order and pseudo-second-order adsorption kinetic models, respectively [50]. The fitted pseudo-second-order results and fitted pseudo-first-order results are shown in Figures S3 and S4, respectively, and the associated model fitting parameters are summarized in Table 1. According to the pseudo-first-order model fitting results, the theoretical adsorption amounts of samples Cit-ACP-25, Cit-ACP-200, Cit-ACP-400, and Cit-ACP-600 were 8.11, 6.78, 7.19, and 9.28 mg/g, respectively. Based on the pseudo-second-order model fitting results, the theoretical adsorption amounts of samples Cit-ACP-25, Cit-ACP-200, Cit-ACP-400, and Cit-ACP-600 were 12.38, 10.38, 9.70, and 2.94 mg/g, respectively. By comparing the correlation coefficients R^2^ of the two models, the R^2^ values of the pseudo-second-order model are higher, and the theoretical adsorption amounts calculated by the pseudo-second-order kinetic model are closer to those obtained from the experiments, which indicates that the pseudo-second-order kinetic model can better describe the adsorption kinetic behavior of the samples.

#### 3.2.2. Adsorption Isotherms

**Figure 5:**
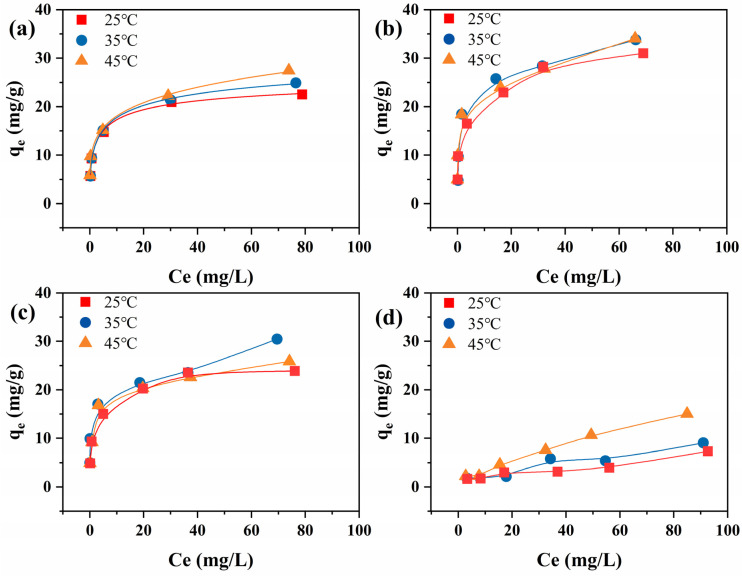
Adsorption isotherms of fluoride ions on the samples at different temperatures (adsorbent dose: 1.0 g/L; time: 24 h; pH: 7.0; initial fluoride ion concentration range: 5–100 mg/L). (**a**) Cit-ACP-25, (**b**) Cit-ACP-200, (**c**) Cit-ACP-400, and (**d**) Cit-ACP-600.

Then, in order to investigate the adsorption capacity of the samples, adsorption isothermal experiments were carried out as shown in Figure 5. The experimental data were linearly fitted using Langmuir model (Figure S5) and Freundlich model (Figure S6), respectively. The calculated parameters of Langmuir and Freundlich models are listed in Table 2.

By comparing the linear fit correlation coefficients (R^2^) of the two models, it was found that the adsorption behavior of the prepared samples was closer to that of the Langmuir model, suggesting that the adsorption behavior of the samples should be a monomolecular layer adsorption. The adsorption capacity of the prepared samples increased with increasing temperature and the initial concentration of fluoride ions. The maximum adsorption capacity (q_m_) of samples Cit-ACP-25, Cit-ACP-200, Cit-ACP-400, and Cit-ACP-600 reached 27.48, 34.05, 25.86, and 15.06 mg/g at an initial fluorine concentration of 100 mg/L and a temperature of 45 °C, respectively.

For comparison, the q_m_ values of fluoride ions on the adsorbents of various calcium phosphate compounds are presented in Table 3. It can be seen that sample Cit-ACP-25 has a higher adsorption capacity than most of the calcium phosphate compound adsorbents reported in the literature.

#### 3.2.3. Adsorption Thermodynamic Parameters Analysis

Usually, whether an adsorption process is absorptive or exothermic or spontaneous or non-spontaneous can be determined by calculating its thermodynamic parameters. The thermodynamic parameters include standard enthalpy change (ΔH), standard entropy change (ΔS), and standard free energy change (ΔG), which can be calculated based on adsorption isotherm data at different temperatures.

The ΔG can be calculated from the following equation:

(7) ΔG=−RTlnK

where *K* is the equilibrium constant in L/g, *T* is the absolute temperature in *K*, and *R* is the ideal gas constant in J/(mol·K). The value of ln*K* is obtained by first plotting lnKd (distribution coefficient *K_d_* = *q_e_*/*C_e_*) versus Ce (Figure S7) and then extrapolating *C_e_* to zero, with the value of the y-axis intercept being the value of ln*K*.

The Δ*H* and Δ*S* were calculated from a linear plot (Figure S8) of ln*K* versus 1/T for fluoride adsorption on the samples using the following formula:

(8) $$\ln K=-\frac{\Delta H}{R}[\frac{1}{T}]+\frac{\Delta S}{R}$$

The calculated thermodynamic parameters obtained for the samples during adsorption are listed in Table 4.

The ΔH values of samples Cit-ACP-25, Cit-ACP-200, Cit-ACP-400, and Cit-ACP-600 are positive, indicating that the adsorption process is a heat absorption reaction. The ΔG values of samples Cit-ACP-25, Cit-ACP-200, and Cit-ACP-400 are negative, indicating that the adsorption process was spontaneous. While the ΔG value of samples Cit-ACP-600 is positive, indicating that the adsorption process was non-spontaneous. With the increase in adsorption temperature, the ΔG values of all samples decreased, indicating that the increase in temperature increased the driving force of the adsorption process, which was favorable for the adsorption of fluoride ions on the surface of the samples.

To further demonstrate the fact that physical or chemical adsorption, the value of activation energy (E_a_) and the sticking probability (S*) were estimated using a modified Arrhenius equation based on the experimental data [60]:

(9) $$\theta =1-\frac{C_{e}}{C_{0}}$$

(10) $$\ln(1-\theta )=\ln S^{*}+\frac{E_{a}}{RT}$$

where *θ* is the surface coverage, *C_e_* is the equilibrium concentration, *C*_0_ is the initial concentration, and R is the gas constant.

The values of S* of fluoride ions adsorption onto samples were 0, 0, 0, and 0.3, respectively, indication that the adsorption was a chemical adsorption. The activation energies of samples Cit-ACP-25, Cit-ACP-200, Cit-ACP-400, and Cit-ACP-600 were calculated as 33.57 kJ·mol^−1^, 69.34 kJ·mol^−1^, 87.21 kJ·mol^−1^, and 2.57 kJ·mol^−1^, respectively, from the curve of ln(*C_e_*/*C_0_*) versus 1/T (Figure S9), demonstrating that the samples Cit-ACP-25, Cit-ACP-200, and Cit-ACP-400 were characteristic of chemical adsorption.

#### 3.2.4. The Effect of pH and Co-Existing Ions

In practice, during the adsorption process for the treatment of fluoride-contaminated water, the performance of the adsorbent is affected by the pH value of the water and the co-existing ions in the water.

**Figure 6:**
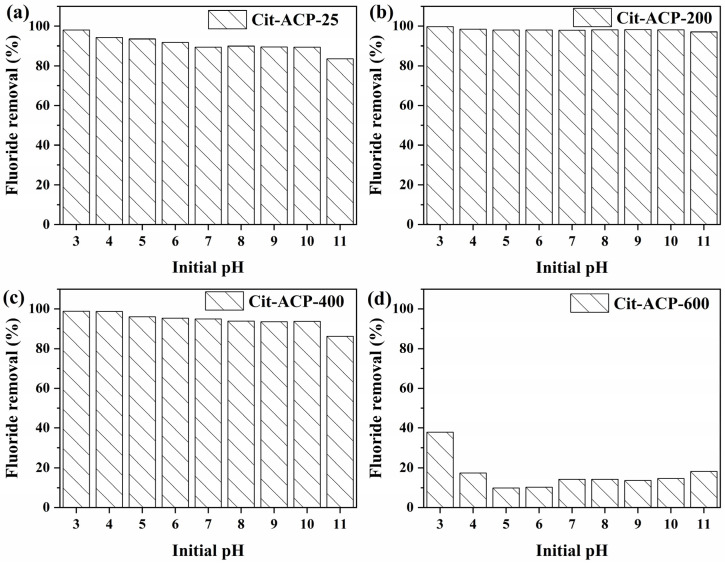
Effect of initial pH on fluoride adsorption (adsorbent dose: 1 g/L; C_0_: 10 mg/L; adsorption temperature: 25 °C; adsorption time: 24 h; initial pH of fluoride solution: 7.0). (**a**) Cit-ACP-25, (**b**) Cit-ACP-200, (**c**) Cit-ACP-400, and (**d**) Cit-ACP-600.

Figure 6 shows the effect of pH on the fluoride removal efficiency of the samples, where the pH ranges from 3 to 11. Overall, the adsorption performance of all the samples decreased as the pH increased from 3 to 11. The decreases in samples Cit-ACP-25, Cit-ACP-200, and Cit-ACP-400 were small, e.g., the decreases in the removal rates were only 3–5% in the pH range from 3 to 11, indicating that the samples Cit-ACP-25, Cit-ACP-200, and Cit-ACP-400 could maintain high fluoride removal efficiencies over a wide range of pH values. However, the adsorption properties of sample Cit-ACP-600 were sensitive to pH, e.g., the removal rate was 37.88% at pH 3 and 9.89% at pH 5.

This relationship between adsorption efficiency and solution pH may be related to the citrate ions adsorbed on the surface of the nanoparticles. For samples calcined at low temperatures (<600 °C), the surface of the particles binds to a large number of citrate ions or partially decomposed citrate, resulting in a buffering effect on the solution pH. In contrast, for the sample calcined at 600 °C, the adsorbed citrate ions on the surface of the particles needed to be completely decomposed and dissipated in order to have a buffering effect on the solution pH [44,61].

**Figure 7:**
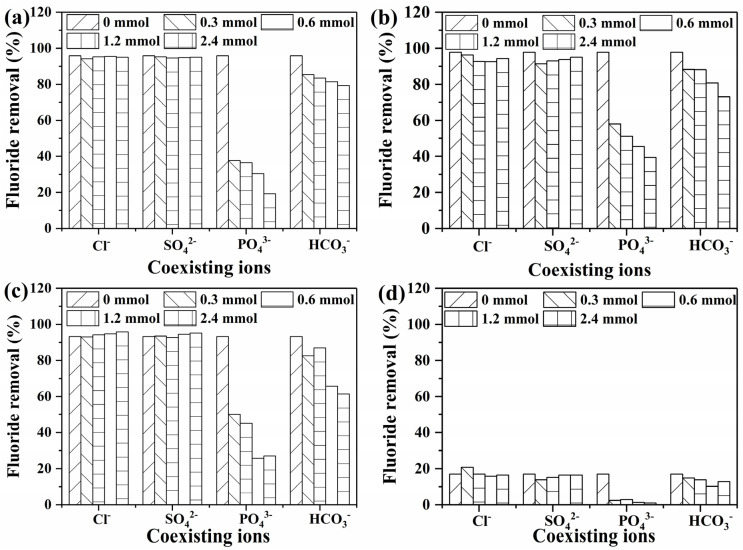
Effect of co-existing anions on fluoride adsorption (adsorbent dose: 1 g/L; C_0_: 10 mg/L; adsorption temperature: 25 °C; adsorption time: 24 h; initial pH of fluoride solution: 7.0). (**a**) Cit-ACP-25, (**b**) Cit-ACP-200, (**c**) Cit-ACP-400, and (**d**) Cit-ACP-600.

Figure 7 shows the effect of co-existing anions on the fluoride removal performance of the samples. The removal of fluoride by the prepared samples was almost the same as that of the blank samples, which was around 95% in the studied concentration ranges of chloride and sulfate ions. When the bicarbonate ion concentration in water was increased from 0 mM to 2.4 mM, the fluoride removal efficiencies of samples Cit-ACP-25, Cit-ACP-200, Cit-ACP-400, and Cit-ACP-600 decreased from 92.82%, 97.78%, 93.25%, and 17.09% to 79.26%, 73%, 61.34%, and 12.91%, respectively. When the phosphate ion concentration in the water was increased from 0 mM to 2.4 mM, the fluoride removal efficiency of samples Cit-ACP-25, Cit-ACP-200, Cit-ACP-400, and Cit-ACP-600 decreased from 92.82%, 97.78%, 93.25%, and 17.09% to 19.25%, 39.4%, 27.08%, and 0.98%, respectively.

This indicates that both phosphate and bicarbonate ions have a significant reducing effect on the fluoride removal efficiency of the samples in the concentration range studied, especially phosphate ions. The effect of the presence of phosphate and bicarbonate on the fluoride removal efficiency of the samples may be mainly attributed to the fact that hydroxide ions are released from the hydrolysis of both anions in water, resulting in an increase in the pH of the water, which affects the charging of the solid surface of the adsorbent, especially when the pH of the water is higher than the isoelectric point of the solid surface. Secondly, phosphate ions also tend to combine with calcium ions on the solid surface, occupying adsorption sites and competing with fluoride ions for adsorption.

#### 3.2.5. Adsorption Mechanism

In the previous section, we systematically evaluated the adsorption properties of the prepared samples. From this, we can see that amorphous calcium phosphate shows better adsorption performance compared to hydroxyapatite. The mechanism of fluoride removal by hydroxyapatite is commonly reported, but the mechanism of fluoride removal by ACP is rarely reported. In order to investigate the fluoride removal mechanism of ACP, we compared the changes in Cit-ACP-25 before and after adsorption.

**Figure 8:**
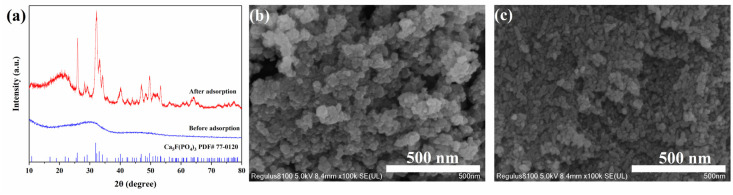
XRD patterns (**a**) and SEM images of sample Cit-ACP-25 before (**b**) and after (**c**) fluoride adsorption.

The XRD spectra and particle morphology changes in sample Cit-ACP-25 before and after adsorption are given in Figure 8. Before adsorption, the XRD spectra (Figure 8a) of sample Cit-ACP-25 had no observable characteristic diffraction peaks, indicating that sample Cit-ACP-25 was amorphous before adsorption [35]. After adsorption, the diffraction pattern of Cit-ACP-25 changed significantly with diffraction peaks at 26.00°, 32.07°, 33.23°, 34.28°, 47.03°, and 49.75°, which corresponded to the characteristic peaks <002>, <211>, <300>, <102>, < 222>, and <213> of the Fluorapatite Standard Card (PDF # 77-0120). Other than this, no other observable characteristic peaks were present. This indicates that Cit-ACP-25 is converted from amorphous calcium phosphate to fluorapatite in the process of fluoride removal and no other products, such as calcium fluoride, appear [62].

It has been reported that the rapid conversion of amorphous calcium phosphate to fluorapatite can be achieved under mild conditions with the co-existence of fluoride ions and citrate ions, which suggests that amorphous calcium phosphate is sensitive to fluoride ions and is susceptible to immobilization by amorphous calcium phosphate [44]. Figure 8b,c shows the changes in particle morphology before and after adsorption of Cit-ACP-25. Before adsorption (Figure 8b), Cit-ACP-25 was mainly in the form of spherical particles of uniform size with an average diameter of 33 nm. After adsorption (Figure 8c), Cit-ACP-25 still maintained a uniform spherical shape, but the average diameter decreased to 18 nm. This may be attributed to the size contraction of amorphous calcium phosphate during fluoride ion adsorption by transforming it into fluorapatite and releasing the structural water from the original molecular structure [63].

**Figure 9:**
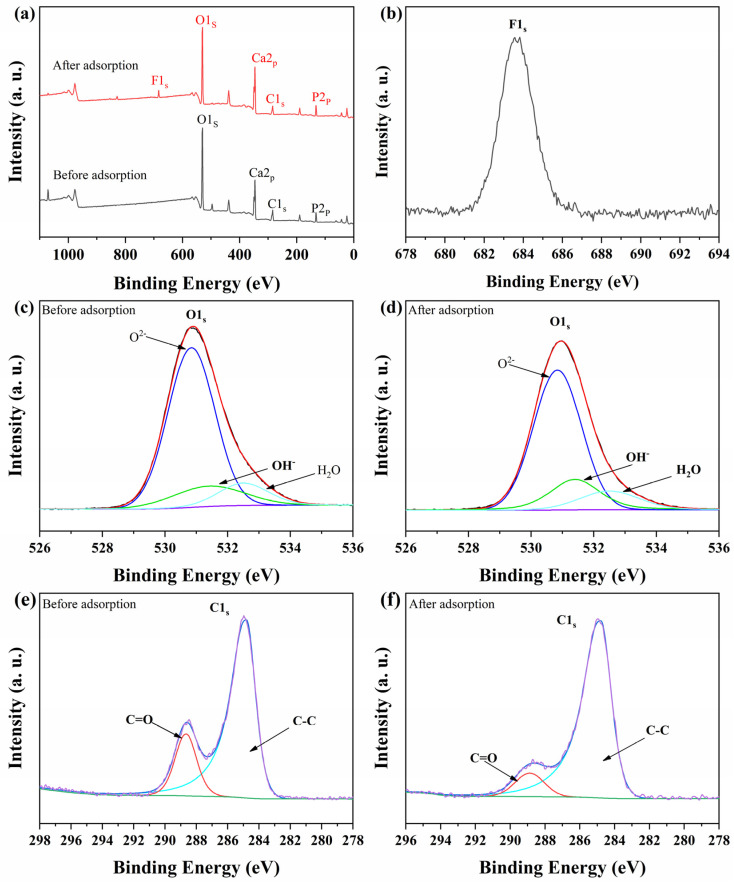
(**a**) XPS spectra of Cit-ACP-25 before and after defluorination; (**b**) F1s spectra of Cit-ACP-25 after defluorination; (**c**) O1s spectra of Cit-ACP-25 before defluorination; (**d**) O1s spectra of Cit-ACP-25 after defluorination; (**e**) C1s spectra of Cit-ACP-25 before defluorination; (**f**) C1s spectra of Cit-ACP-25 after defluorination.

Figure 9 shows the XPS patterns of Cit-ACP-25 before and after the adsorption of fluoride ions. As can be seen from the total spectrum of Figure 9a, before adsorption, there were no fluorine F1s peaks present on the spectrum, while fluorine F1s peaks clearly appear on the sample after adsorption, which indicates that fluorine ions are adsorbed within the material after defluorination of the two adsorbents. Figure 9b exhibits the F1s peak after adsorption of Cit-ACP-25, which further proves the presence of fluoride ions.

Figure 9c,d represent the O1s peak splitting before and after the adsorption of fluoride ions by Cit-ACP-25, and the specific data of the peak splitting are shown in Table 5. The O1s profile can be divided into three peaks, which are O^2−^ (530.5 eV), OH^−^ (531.3 eV), and H_2_O (532.5 eV) [50,64]. From Table 5, it can be seen that the content of O^2−^ of Cit-ACP-25 remained almost unchanged, while the H_2_O decreased from 20.2% to 18.4%, and the OH^−^ increased from 30.8% to 32.3%. This may be due to the crystallization of Cit-ACP during defluoridation, which releases structural water and forms the more thermodynamically stable fluorapatite or hydroxyapatite.

Figure 9e,f represent the C1s peak splitting before and after the adsorption of fluoride ions by Cit-ACP-25, and the specific data of the peak splitting are shown in Table 6. The C1s profile can be divided into two peaks, which are C-C (284.8 eV) and O-C=O (288.64 eV) [65]. From Table 6, it can be seen that the content of the O-C=O decreased from 20.4% to 10.74%, the content of the C-C increased from 79.6% to 89.26%. This may be due to the release of surface adsorbed citrate molecules during the crystallization of Cit-ACP.

**Figure 10:**
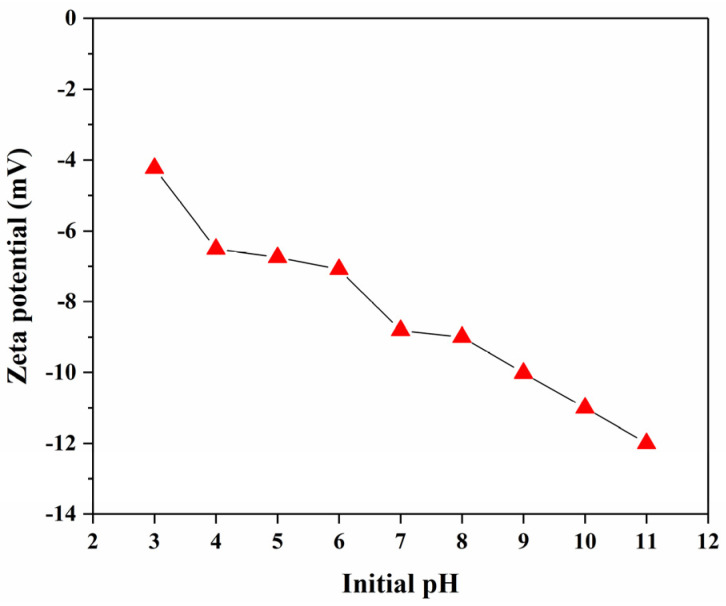
Zeta potential of Cit-ACP-25 nanoparticles under different initial pH conditions (adsorbent dose: 1.0 g/L; initial fluoride ion concentration: 10 mg/L; temperature: 25 °C; time: 24 h).

Figure 10 shows the variation in zeta potential values of Cit-ACP-25 nanoparticles at different pHs. The surface of Cit-ACP-25 nanoparticles is negatively charged in the pH range studied. The absolute zeta potential of Cit-ACP-25 gradually increases from 5 mV to 10 mV as the initial pH of solution increases from 3 to 11, indicating a gradual increase in the charge on the surface of the particles. Even so, Cit-ACP-25 nanoparticles are still weakly negative in solution. Fluoride ions are negatively charged in aqueous solution. If the initial pH is maintained at neutral or weakly acidic, it is favorable to reduce the electrostatic repulsive force and enhance the rapid binding of fluoride ions with the adsorbent. If the initial pH value is maintained at weak base or alkaline, it will increase the electrostatic repulsion force, making it difficult for fluoride ions to approach the solid surface of the adsorbent, resulting in a decrease in the efficiency of fluoride removal.

**Figure 11:**
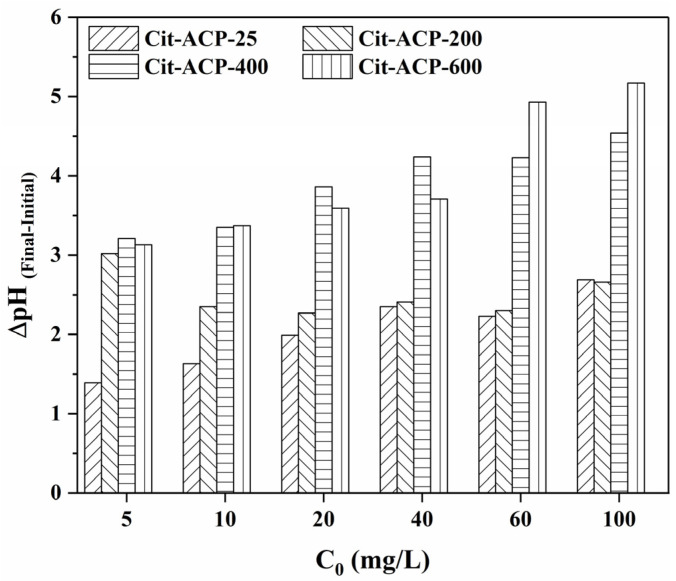
pH variation in the solution before or after fluoride adsorption.

Figure 11 exhibits the change in pH of the solution before and after the adsorption. Overall, the pH of the solution after adsorption was higher than before adsorption for all the samples. There are also two points of concern. The first is that the pH difference before and after adsorption increases as the initial concentration of the fluoride ion solution increases. The second is that the pH difference in samples Cit-ACP-400 and Cit-ACP-600 is much larger than that of samples Cit-ACP-25 and Cit-ACP-200.

These changes in solution pH may be attributed to two causes. One, for samples Cit-ACP-25 and Cit-ACP-200, the adsorption of fluoride ions on the surface of the samples converted the samples from ACP to fluorapatite, which resulted in the desorption of sodium citrate molecules adsorbed on the surface of the samples into the solution, resulting in an increase in the solution pH. Second, for the samples Cit-ACP-400 and Cit-ACP-600, since they are mainly hydroxyapatite, the adsorption of fluoride ions in the samples is mainly attributed to ion exchange with hydroxide ions in the crystals, resulting in the entry of hydroxide ions into the solution, causing an increase in solution pH [50].

**Figure 12:**
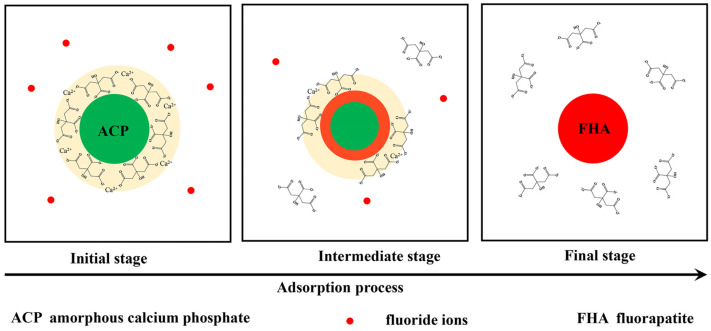
Proposed adsorption mechanism of sample Cit-ACP-25.

Based on the above analysis of the sample Cit-ACP-25 before and after the adsorption of fluoride ions, the adsorption mechanism may be inferred as shown in Figure 12.

The XRD results demonstrate that Cit-ACP-25, prepared via sodium citrate-assisted chemical precipitation at room temperature, exhibits a characteristic amorphous phase. Remarkably, this amorphous nature persists even after calcination at 200 °C, indicating the strong stabilizing effect of sodium citrate on amorphous calcium phosphate [43,66]. SEM and BET characterization reveal that Cit-ACP-25 consists of spherical nanoparticles with an average diameter of 33 nm and a specific surface area of 113.32 m^2^/g. Notably, this high surface area was achieved through a simple room-temperature preparation process without complex procedures, representing a significant advantage over previously reported methods in the literature [2]. The adsorption performance of the Cit-ACP-25 sample could be well described by the Langmuir model, and the adsorption kinetic followed the pseudo-second-order model. The maximum adsorption capacity was 27.48 mg/g at pH 7.0 when the fluoride concentration was 100 mg/L. The thermodynamic parameters suggested that the adsorption of fluoride was a spontaneous endothermic process. Comparative studies reveal that Cit-ACP-25 exhibits superior adsorption performance over most reported calcium phosphate-based adsorbents [67,68]. Notably, its adsorption capacity also significantly surpasses that of its calcined counterparts. By analyzing the physicochemical changes in Cit-ACP-25 before and after adsorption, we elucidate the underlying adsorption mechanisms.

Unlike conventional hydroxyapatite adsorption through the mechanism of ion exchange between fluoride ions and hydroxide ions on the surface of hydroxyapatite crystals, Cit-ACP nanoparticles are spherical with a core–shell structure, consisting of an amorphous calcium phosphate as the core and a calcium citrate adsorption layer as the shell. When the Cit-ACP nanoparticles come into contact with the fluoride ions in the solution, the fluoride ions pass through the shell layer into the surface of the ACP core and come into contact with the amorphous calcium phosphate, which leads to the rapid conversion of ACP to fluorapatite. The fluorapatite transformation process proceeds from surface to surface, with the ACP in the inner core gradually and completely transformed into fluorapatite. During the transformation process, calcium ions from calcium citrate in the shell layer are involved in the transformation of fluorapatite by ACP, while sodium citrate is formed and released into solution. This is why Cit-ACP is insensitive to the pH of the adsorption environment and can still maintain a high adsorption efficiency over a wide pH range.

The possible chemical reaction pathways are listed below:

3[Ca_3_(PO_4_)_2_·yCa_3_(Cit)_2_] + 2NaF = Ca_10_(PO_4_)_6_F_2_ + 2/3Na_3_Cit + (3y − 1/3)Ca_3_(Cit)_2_ (11)

where Ca_3_(PO_4_)_2_·yCa_3_(Cit)_2_ is the Cit-ACP.

## 4. Conclusions

In conclusion, Cit-ACP nanoparticles were successfully prepared at room temperature based on sodium citrate-assisted chemical synthesis method, while the particles were sintered at different temperatures for comparison. It was found that the products prepared at room temperature were pure amorphous calcium phosphate with high specific surface areas and that the sintering treatment reduced the specific surface areas of the products and resulted in the gradual transformation from amorphous calcium phosphate to hydroxyapatite. Fluoride ion adsorption performance evaluation revealed that Cit-ACP had a high fluoride ion adsorption capacity of 27.48 mg/g at an initial fluoride ion concentration of 100 mg/L at 45 °C. Isothermal adsorption studies and adsorption kinetic studies showed that the fluoride ion adsorption behavior of Cit-ACP followed the Langmuir adsorption isothermal model and the pseudo-second-order kinetic model. It was also found that Cit-ACP has a wide pH and co-existing ion adsorption stability. By comparing the changes before and after the adsorption of fluoride ions on Cit-ACP, the main adsorption mechanism is that fluoride ions and calcium ions in the shell layer of Cit-ACP promote the transformation of amorphous calcium phosphate into fluorapatite.

## Figures and Tables

**Table 1:** Parameters for pseudo-first-order and pseudo-second-order kinetic models of fluoride adsorption on the prepared samples.

Sample	C_0_ (mg/L)	q_e(exp)_ (mg/g)	Pseudo-First-Order		Pseudo-Second-Order			
			K_1_·10^−3^	q_e(cal)_	R^2^	K_2_·10^−3^	q_e(cal)_	R^2^
Cit-ACP-25	10	9.50	2.97	8.11	0.852	0.26	12.38	0.980
Cit-ACP-200		9.78	2.48	6.78	0.903	0.92	10.38	0.993
Cit-ACP-400		9.32	1.69	7.19	0.952	0.84	9.70	0.990
Cit-ACP-600		1.71	0.39	9.28	0.661	0.63	2.94	0.771

**Table 2:** Parameters for Langmuir and Freundlich models of fluoride adsorption on samples.

Sample	T (°C)	Langmuir Constants				Freundlich Constants		
		q_m(cal)_ (mg/g)	q_m(exp)_ (mg/g)	K_L_ (L/mg)	R^2^	K_F_ (mg^1n^·L^n^/g)	*n*	R^2^
Cit-ACP-25	25	22.87	22.51	0.576	0.999	9.10	4.31	0.948
	35	25.25	24.89	0.427	0.995	9.26	4.74	0.957
	45	27.69	27.48	0.400	0.988	11.22	4.82	0.983
Cit-ACP-200	25	32.03	30.99	0.285	0.996	10.18	3.66	0.947
	35	33.55	33.80	0.541	0.995	14.26	4.56	0.934
	45	34.04	34.05	0.34	0.992	11.30	3.65	0.929
Cit-ACP-400	25	24.41	23.87	0.50	0.998	9.07	3.90	0.955
	35	29.74	30.47	0.34	0.987	12.72	5.12	0.904
	45	25.91	25.86	0.41	0.996	9.64	4.00	0.939
Cit-ACP-600	25	8.13	7.35	0.03	0.846	0.84	2.39	0.843
	35	12.40	9.08	0.02	0.802	0.76	1.96	0.803
	45	23.83	15.06	0.017	0.876	0.91	1.63	0.920

**Table 3:** Comparison of fluoride adsorption capacity on various calcium phosphate compound adsorbents at 100 mg/L initial fluoride concentration.

Adsorbents	q_e_ (mg/g)	pH	Ref.
Sulfate-doped hydroxyapatite	28.3	7	[17]
Marble apatite	7.2	7	[51]
Cerium-containing bone char	9.1	7	[52]
Al-doped cow bone char	18.5	7	[53]
Non-calcined synthetic hydroxyapatite	7.5	5	[54]
Serpentine-loaded hydroxyapatite	5.7	7	[55]
Hydroxyapatite nanowires	29.8	7	[50]
Ag/MgO/HAP nanoparticles	6.9	7	[56]
Montmorillonite/hydroxyapatite nanocomposite	16.7	5	[57]
Hydroxyapatite decorated with carbon nanotube	11.05	6	[58]
Cubical-shaped rods of pectin–hydroxyapatite composite	28.57	7	[59]
Cit-ACP	27.48	7	This study

**Table 4:** The thermodynamic parameters for the adsorption of fluoride ions in water by samples.

Sample	Temp. (K)	∆G (kJ/mol)	∆H (kJ/mol)	∆S (J/mol·K)
Cit-ACP-25	298	−4.32	24.87	97.18
	308	−4.56		
	318	−6.29		
Cit-ACP-200	298	−5.96	49.21	184.21
	308	−6.92		
	318	−9.68		
Cit-ACP-400	298	−5.49	50.24	185.69
	308	−6.32		
	318	−8.96		
Cit-ACP-600	298	3.22	19.62	54.55
	308	3.13		
	318	2.11		

**Table 5:** O(1s) peak parameters of the Cit-ACP-25 before and after fluoride adsorption.

Sample	Peak	BE (eV)	Percent (%)
Cit-ACP-25	O^2−^	530.5	49.1
	OH^−^	531.3	30.8
	H_2_O	532.5	20.2
Cit-ACP-F	O^2−^	530.3	49.2
	OH^−^	531.3	32.3
	H_2_O	532.3	18.4

**Table 6:** C(1s) peak parameters of the Cit-ACP-25 before and after fluoride adsorption.

Sample	Peak	BE (eV)	Percent (%)
Cit-ACP-25	C-C	284.8	79.6
	O-C=O	288.64	20.4
Cit-ACP-F	C-C	284.8	89.26
	O-C=O	288.88	10.74

## Data Availability

Data are contained within the article and Supplementary Materials.

## Supplementary Materials

The following supporting information can be downloaded at: https://www.mdpi.com/article/10.3390/nano15080621/s1, Figure S1: Schematic illustration of samples preparation; Figure S2: Standard curve of linear relationship between fluoride ion standard concentration and electrode potential, (pH = 7.0, Y = 60.13X − 12.36, R^2^ = 0.999); Figure S3: The pseudo-first-order kinetic plots for the adsorption of fluoride ions on the prepared samples, (a) Cit-ACP-25, (b) Cit-ACP-200, (c) Cit-ACP-400, (d) Cit-ACP-600. (adsorbent dose: 1.0 g/L, temperature: 25 °C, pH:7.0); Figure S4: The pseudo-second-order kinetic plots for the adsorption of fluoride ions on the prepared samples, (a) Cit-ACP-25, (b) Cit-ACP-200, (c) Cit-ACP-400, (d) Cit-ACP-600. (Adsorbent dose: 1.0 g/L, temperature: 25 °C, pH:7.0); Figure S5: Langmuir isotherm models for fitting of fluoride adsorption on the prepared samples, (a) Cit-ACP-25, (b) Cit-ACP-200, (c) Cit-ACP-400, (d) Cit-ACP-600. (Adsorbent dose: 1.0 g/L, time: 24 h, pH: 7.0); Figure S6: Freundlich isotherm models for fitting of fluoride adsorption on the prepared samples, (a)Cit-ACP-25, (b) Cit-ACP-200, (c) Cit-ACP-400, (d) Cit-ACP-600. (Adsorbent dose: 1.0 g/L, time: 24 h, pH: 7.0); Figure S7: Linear fits of lnKd and Ce for the adsorption of the samples for different fluoride ion concentrations at different temperatures, (a) Cit-ACP-25, (b) Cit-ACP-200, (c) Cit-ACP-400, (d) Cit-ACP-600; Figure S8: Linear fits of lnK0 and 1/T for the samples, (a) Cit-ACP-25, (b) Cit-ACP-200, (c) Cit-ACP-400, (d) Cit-ACP-600; Figure S9: Linear fits of ln(1-θ) and 1/T for the samples, (a) Cit-ACP-25, (b) Cit-ACP-200, (c) Cit-ACP-400, (d) Cit-ACP-600.
