# Supplementary material for: Citrate-Stabilized Amorphous Calcium Phosphate Nanoparticles as an Effective Adsorbent for Defluorination
Source: Nanomaterials (Basel). 2025 Apr 18;15(8):621. doi: 10.3390/nano15080621 (PMC12029190; doi:10.3390/nano15080621)
Supplement: Supplementary file 1 [file nanomaterials-15-00621-s001.zip › nanomaterials-3526910-supplementary.pdf]

Supporting information

Citrate-stabilized amorphous calcium phosphate nanoparticles as an effective adsorbent for defluorination

Ruojiao Su *, Miaomiao Wang, Yuwei Jiang, Junjun Tan *

Corresponding author (*):

Junjun Tan;

Affiliations:

Hubei Province Key Laboratory of Green Materials for Light Industry, Collaborative Innovation Center for Green Lightweight Materials and Processing, Hubei University of Technology, Wuhan 430068, P. R. China

Tel: +86 27 5975 0487

Corresponding author e-mail address: tanjunjun2011@hbut.edu.cn

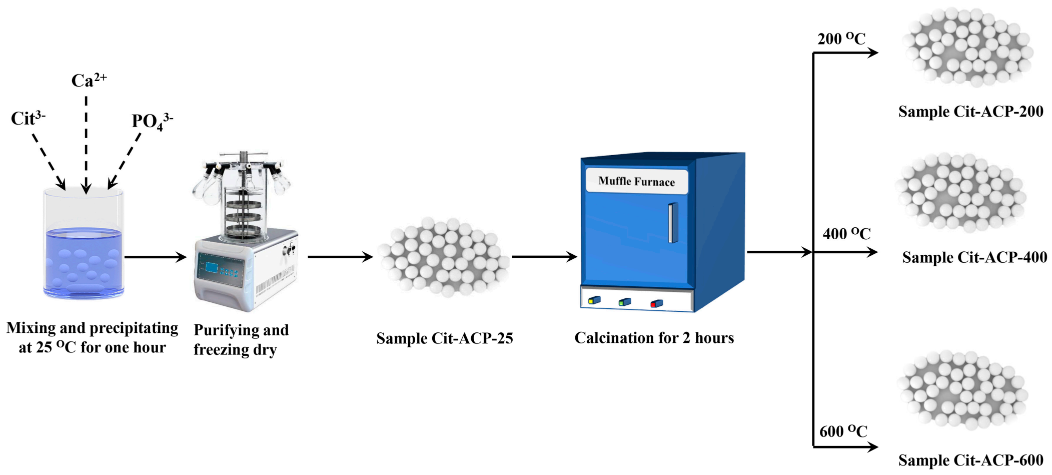

Figure S1. Schematic illustration of samples preparation.

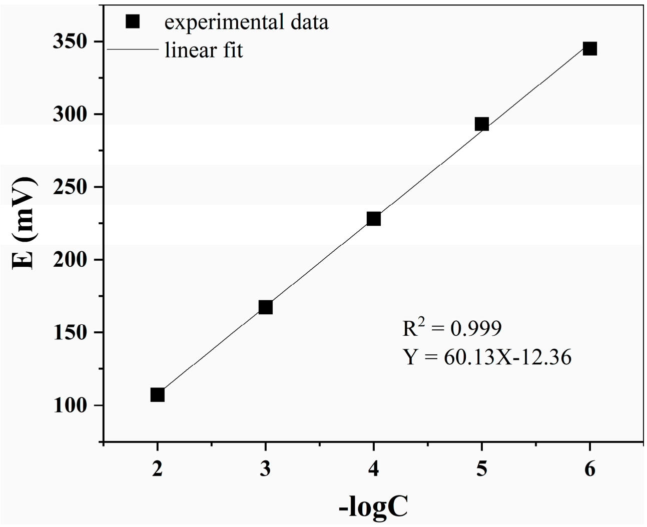

Figure S2. Standard curve of linear relationship between fluoride ion standard concentration and electrode potential, (pH = 7.0, Y = 60.13X – 12.36, R^2 = 0.999).

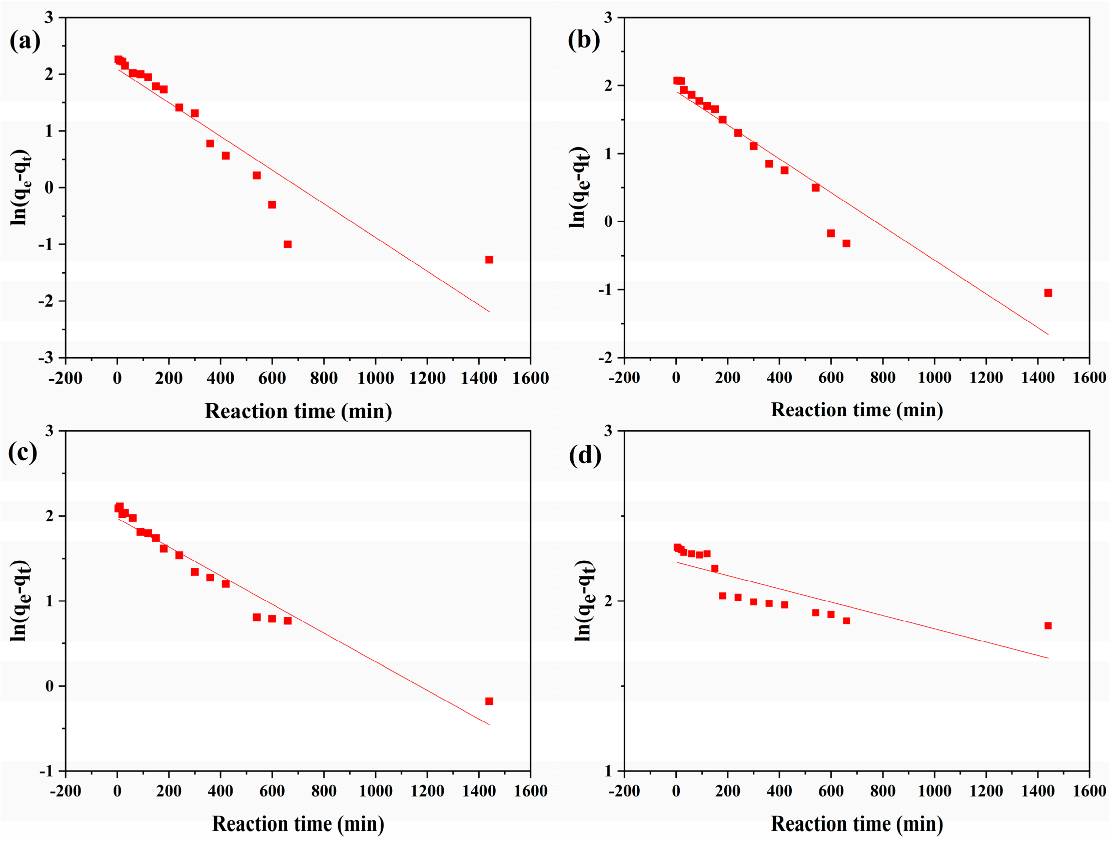

Figure S3. The pseudo-first-order kinetic plots for the adsorption of fluoride ions on the prepared samples, (a) Cit-ACP-25, (b) Cit-ACP-200, (c) Cit-ACP-400, (d) Cit-ACP-600. (adsorbent dose: 1.0 g/L, temperature:25°C, pH:7.0)

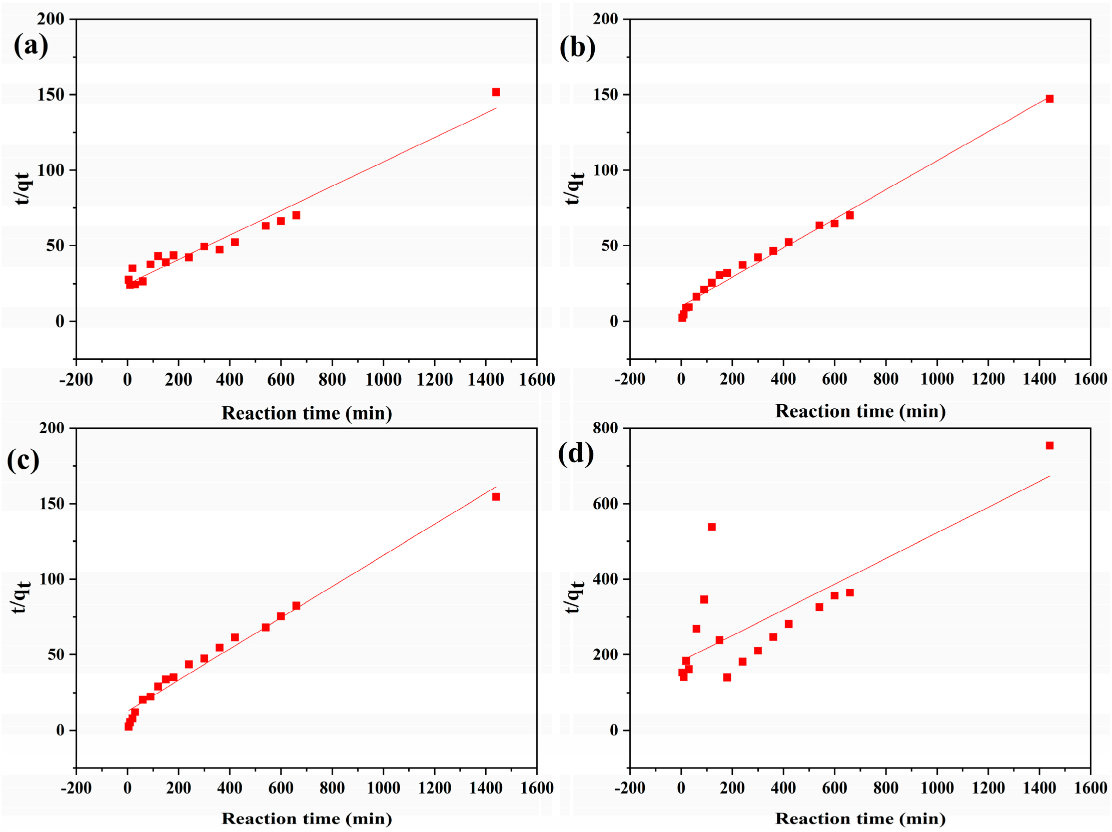

Figure S4. The pseudo-second-order kinetic plots for the adsorption of fluoride ions on the prepared samples, (a) Cit-ACP-25, (b) Cit-ACP-200, (c) Cit-ACP-400, (d) Cit-ACP-600. (Adsorbent dose: 1.0 g/L, temperature:25°C, pH:7.0)

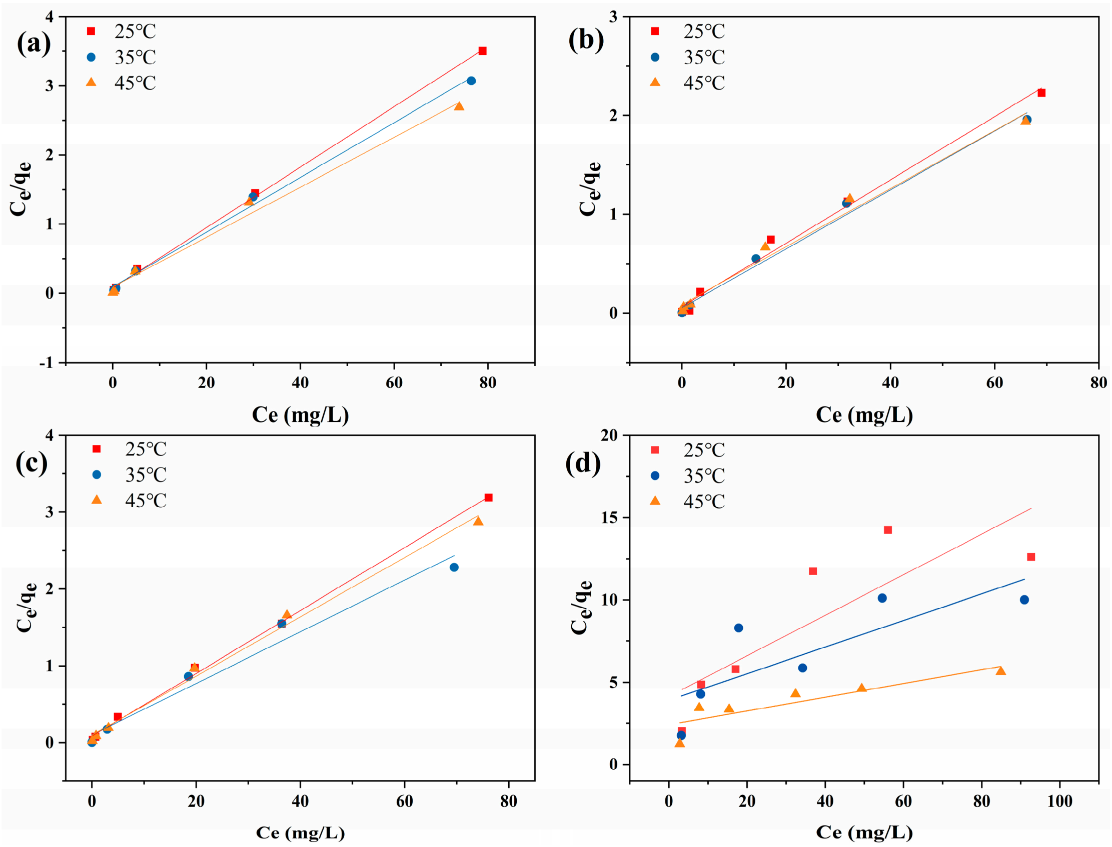

Figure S5. Langmuir isotherm models for fitting of fluoride adsorption on the prepared samples, (a) Cit-ACP-25, (b) Cit-ACP-200, (c) Cit-ACP-400, (d) Cit-ACP-600. (Adsorbent dose: 1.0 g/L, time: 24 h, pH: 7.0)

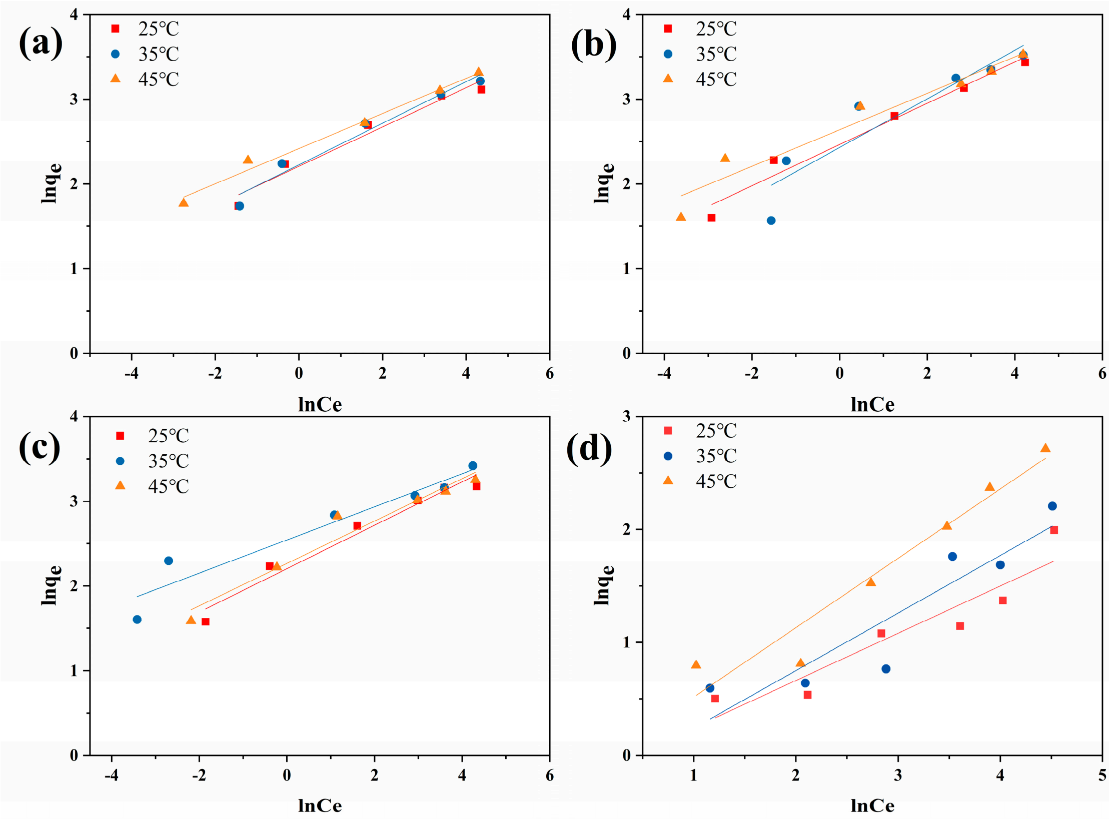

Figure S6. Freundlich isotherm models for fitting of fluoride adsorption on the prepared samples, (a)Cit-ACP-25, (b) Cit-ACP-200, (c) Cit-ACP-400, (d) Cit-ACP-600. (Adsorbent dose: 1.0 g/L, time: 24 h, pH: 7.0)

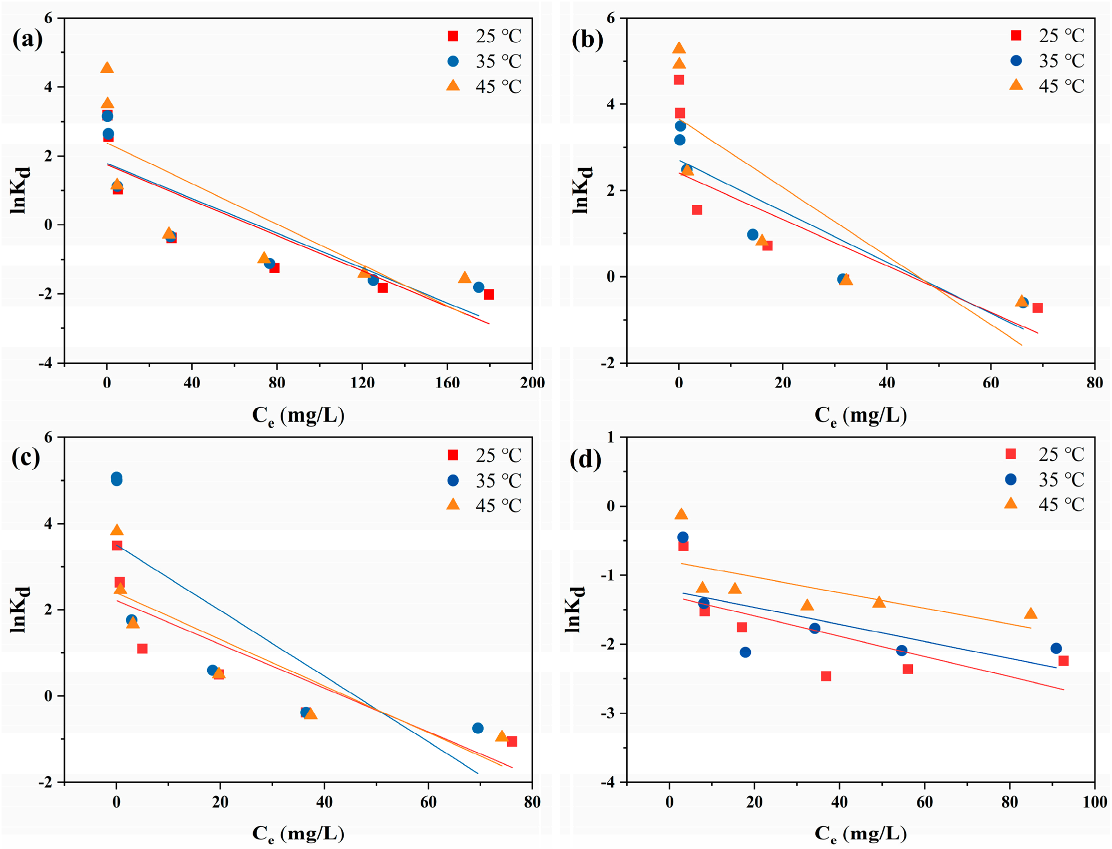

Figure S7. Linear fits of lnKd and Ce for the adsorption of the samples for different fluoride ion concentrations at different temperatures, (a) Cit-ACP-25, (b) Cit-ACP-200, (c) Cit-ACP-400, (d) Cit-ACP-600.

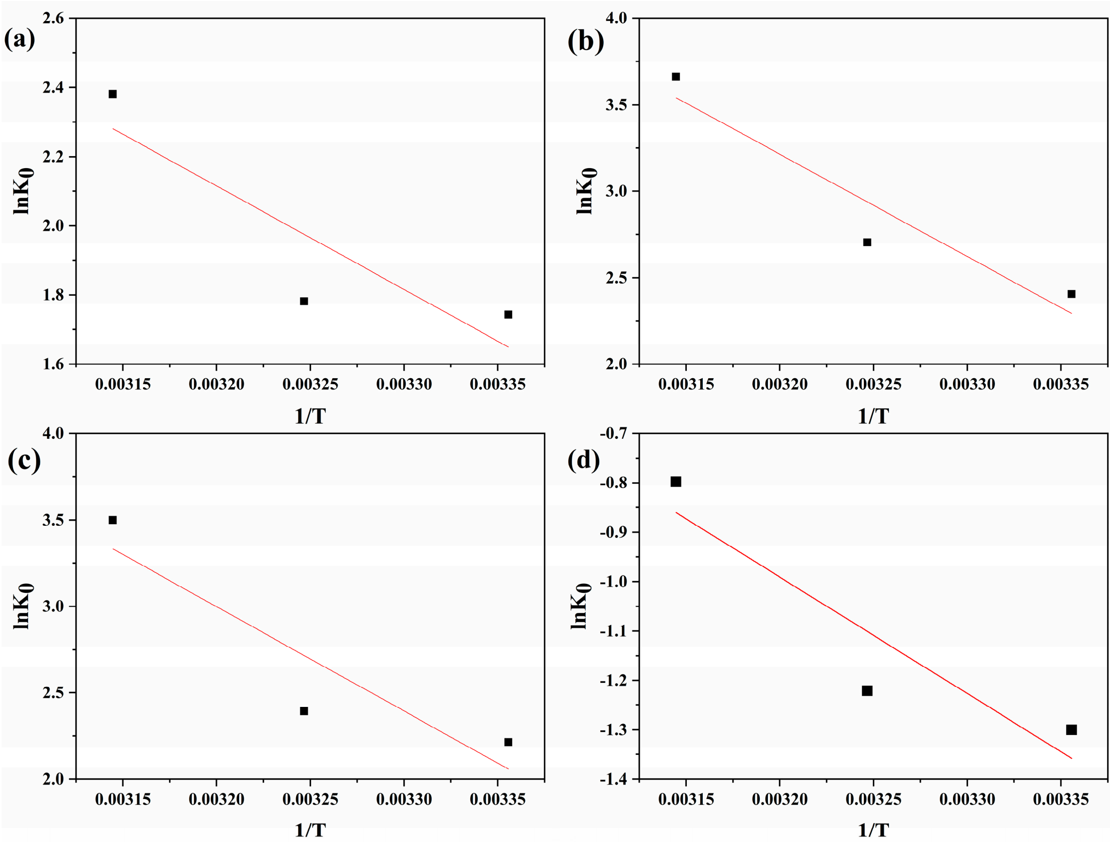

Figure S8. Linear fits of lnK0 and 1/T for the samples, (a) Cit-ACP-25, (b) Cit-ACP-200, (c) Cit-ACP-400, (d) Cit-ACP-600.

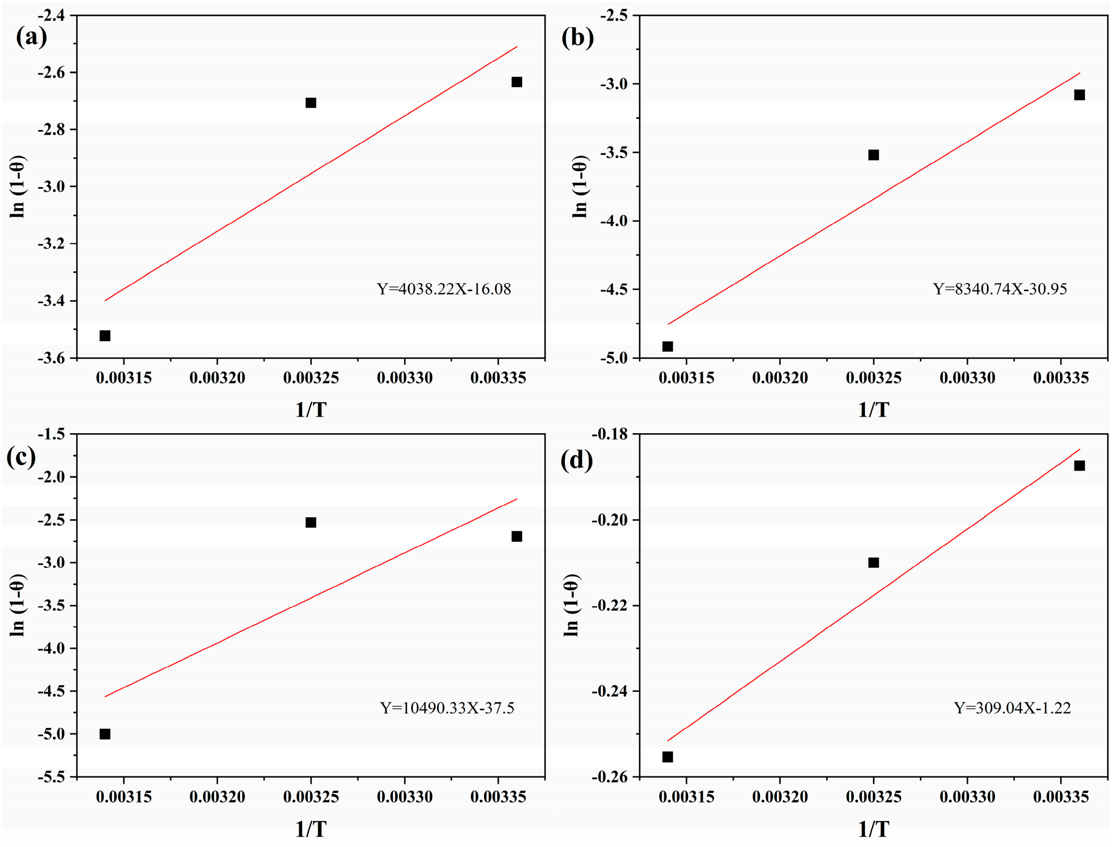

Figure S9. Linear fits of ln(1-θ) and 1/T for the samples, (a) Cit-ACP-25, (b) Cit-ACP-200, (c) Cit-ACP-400, (d) Cit-ACP-600.
